# Pharmacological Significance of Heme Oxygenase 1 in Prostate Cancer

**DOI:** 10.3390/cimb45050273

**Published:** 2023-05-15

**Authors:** Mohamed Ben-Eltriki, Erysa J. Gayle, Noah Walker, Subrata Deb

**Affiliations:** 1 Department of Pharmacology and Therapeutics, Clinical Pharmacology Lab, Max Rady College of Medicine, University of Manitoba, Winnipeg, MB R3E 0T6, Canada; 2 Cochrane Hypertension Review Group, Therapeutic Initiative, University of British Columbia, Vancouver, BC V6T 1Z3, Canada; 3 College of Biomedical Sciences, Larkin University, 18301 N. Miami Avenue, Miami, FL 33169, USA; 4 Department of Pharmaceutical Sciences, College of Pharmacy, Larkin University, Miami, FL 33169, USA

**Keywords:** heme oxygenase 1, prostate cancer, induction, inhibition, inflammation, precancerous, anti-cancerous, oxidative stress

## Abstract

Heme oxygenase 1 (HO-1) is a detoxifying antioxidant microsomal enzyme that regulates inflammation, apoptosis, cell proliferation, and angiogenesis in prostate cancer (PCa). This makes HO-1 a promising target for therapeutic prevention and treatment due to its anti-inflammatory properties and ability to control redox homeostasis. Clinical evidence highlights the possible correlation between HO-1 expression and PCa growth, aggressiveness, metastasized tumors, resistance to therapy, and poor clinical outcomes. Interestingly, studies have reported anticancer benefits mediated by both HO-1 induction and inhibition in PCa models. Contrasting evidence exists on the role of HO-1 in PCa progression and possible treatment targets. Herein, we provide an overview of available evidence on the clinical significance of HO-1 signaling in PCa. It appears that the beneficial effects of HO-1 induction or inhibition are dependent on whether it is a normal versus malignant cell as well as the intensity (major vs. minor) of the increase in HO-1 enzymatic activity. The current literature evidence indicates that HO-1 has dual effects in PCa. The amount of cellular iron and reactive oxygen species (ROS) can determine the role of HO-1 in PCa. A major increase in ROS enforces HO-1 to a protective role. HO-1 overexpression may provide cryoprotection to normal cells against oxidative stress via suppressing the expression of proinflammatory genes, and thus offer therapeutic prevention. In contrast, a moderate increase in ROS can lead to the perpetrator role of HO-1, which is associated with PCa progression and metastasis. HO-1 inhibition by xenobiotics in DNA-damaged cells tilts the balance to promote apoptosis and inhibit PCa proliferation and metastasis. Overall, the totality of the evidence revealed that HO-1 may play a dual role in the therapeutic prevention and treatment of PCa.

## 1. Introduction

Prostate cancer (PCa) is a commonly diagnosed cancer that ranks second in cancer-related deaths in men in the United States [1]. Although the role of androgens has been the most widely studied area and successfully targeted in PCa, enzymes involved in proliferation, survival, and inflammation have been increasingly explored as therapeutic targets. Even though PCa is remediable if detected early, a large number of patients advance to a more complicated stage known as castration-resistant prostate cancer (CRPC) [2]. Anti-apoptotic, angiogenic, proliferative, and pro-inflammatory activities are key to the development of advanced PCa. The inflammatory pathways involving nuclear factor kappa B (NF-κB) and the modulation of nuclear factor erythroid 2-related factor 2 (Nrf2)-mediated anti-inflammatory pathways underlie the molecular mechanisms of PCa [3,4]. Vascular endothelial growth factor (VEGF) is another angiogenic protein that has a critical role in CRPC. The overexpression of VEGF is central to the progression of PCa and has been pharmacologically inhibited in other cancer types [5,6]. Reactive oxygen species (ROS), which are the byproduct of cellular metabolism in oxidative stress, have the ability to damage DNA as well as trigger the overexpression of VEGF [7]. Similarly, ROS can downregulate tumor suppressor genes (e.g., PTEN and p53) in PCa pathogenesis. The ROS-mediated activation of inflammatory pathways in PCa, leading to cell proliferation and cell survival, involves a complex network of proteins [8]. Thus, lowering oxidative stress through antioxidant enzymes is considered an attractive strategy to prevent PCa development. However, cancerous cells can exploit the upregulation of antioxidant enzymes in tumor growth and metastasis.

Heme oxygenase 1 (HO-1) is a stress-inducible heat shock protein microsomal enzyme that helps to degrade heme, a component of hemoglobin. HO-1 catalyzes the conversion of heme to biliverdin, iron, and carbon monoxide (CO) [9]. The enzyme biliverdin reductase then converts biliverdin to bilirubin. HO-1 is expressed in several tissues including the heart, liver, brain, reproductive organs, and kidneys [10]. HO-1 is a stress response protein that is induced in response to cellular stressors such as oxidative stress, inflammation, and hypoxia [11]. The central functions of HO-1 involve the protection of cells and tissues from oxidative stress. It is interesting to note that some of the key functions of HO-1 are mediated through biliverdin, CO, ferrous iron, and the degradation products of heme. Bilirubin, biliverdin, and CO have anti-inflammatory, antiapoptotic, antioxidant, and immunomodulatory effects, whereas ferrous iron has antioxidant and lipid-oxidation-related ferroptosis actions [11]. The pro-surviving and pro-angiogenic functions of HO-1 are contradictory to its anti-inflammatory role. HO-1 has been indicated in multiple conditions including cardiovascular, diabetes, and cancer. However, the functions of HO-1 in PCa are not fully clear. The pro-cancerous and anticancer effects of HO-1 have been highlighted in different PCa research models [12,13,14,15]. The purpose of the current work is to review the pharmacological targeting of HO-1 from the induction and inhibition perspective in PCa. The expression of HO-1 in PCa models and its corresponding role in inflammation and PCa pathogenesis have also been described.

## 2. Literature Search Strategy

The focused literature search for this narrative review was conducted using specific keywords and common electronic databases until 28 February 2023. Different combinations of the following keywords were utilized to obtain articles related to the focus of this manuscript: heme oxygenase 1, prostate cancer, pathogenesis, induction, inhibition, inflammation, precancerous, anti-cancerous, and metastasis. The alternative spellings, variations, and abbreviations of the above keywords were also taken into account. The PubMed, Medline, and Google Scholar electronic databases were included in the literature search. The study includes original research but not conference abstracts or unpublished prints. The titles and abstracts were examined first followed by an examination of the whole text. Only articles that were published in English are included in the current work. Two authors (S.D. and M.B.-E.) independently conducted the literature search, article evaluation, and resource reconciliation. The minor inconsistencies in the literature search between the investigators were examined and reconciled by consensus.

## 3. Expression of HO-1 in PCa Models

Among the three isozymes of heme oxygenase, HO-1 is the inducible form, whereas HO-2 and HO-3 are constitutively expressed [16,17]. HO-1 is strongly upregulated in response to stressful conditions such as oxidative stress and in the presence of excess intracellular heme [11]. HO-2 is expressed in cells which catalyze heme degradation in basal physiological conditions and exhibits a unique role in hypoxic response [18,19,20]. HO-2 is also expressed in prostate tissues and PCa cells [21,22,23,24]. Zhang et al. reported that hypoxia decreases the expression of HO-2 and subsequently increases cellular heme contents in several human cell lines, including two cancer cell lines: HeLa cervical cancer and HepG2 hepatoma cells [25]. Compared to HO-1, to date, HO-2 has been less studied in the perspective of cancer. Ding et al. have shown that HO-2 does express in eight human cancer cell lines and concluded that HO-2 may play a role in regulating HO-1 expression [26]. Interestingly, the knockdown of HO-2 expression increases HO-1 expression [26]. HO-1 is expressed across various cells within the prostate tumor microenvironment and immune cells, including dendritic cells, macrophages, and T-cells [11,13,14,27]. In PCa, HO-1 was found to be localized at the smooth endoplasmic reticulum membrane and in the nucleus [21,28,29]. Clinical studies have reported that HO-1 expression is increased in PCa patients, which correlates with high Gleason grade, increased serum prostate-specific antigen (PSA), resistance to therapy, and poor clinical outcome [21,29,30,31]. It has been shown that overexpression of truncated nuclear HO-1 correlates with PCa growth, aggressiveness, and metastasized tumors [32].

In human PCa cell lines, studies have shown that high HO-1 expression was associated with the inhibition of PCa cell proliferation in vitro in androgen-sensitive (MDA Pca2b and LNCaP) and androgen-insensitive (PC3) PCa cell lines [13]. HO-1 was found to be overexpressed in metastatic PCa in PC3 cells [12] and was detected in DU145 experimental PCa cell lines [13]. In animal models, HO-1 mRNA and proteins were expressed in rat prostate tumors and in surrounding non-malignant prostate tissue as well as in the macrophages [27]. HO-1 has also been detected in PC3 xenograft-positive and prostate tumor-infiltrating macrophages [14,15]. Halin et al. reported that HO-1 is expressed in bone metastases, and positively correlated with the expression of macrophages, suggesting that it can be potentially used as a marker for a subgroup of bone metastases [27]. High HO-1 expression was associated with slow tumor growth and angiogenesis in vivo [13,33]. In the aggressive form of PCa, HO-1 expression was higher in metastatic PCa samples [15]. HO-1 expression increases outside the prostate tumors as a result of the HO-1+ macrophage synthesis, which was correlated with the presence of bone metastases, suggesting that extra tumoral elevation of HO-1 could serve as a potential biomarker for PCa bone metastases [27].

## 4. Biochemical Role of HO-1 in PCa

Heme (pro-oxidative agent) can cause oxidative stress and triggers cell apoptosis by inhibiting the proteasome [34]. HO-1 is a detoxifying antioxidant enzyme, the stress-induced isoform in response to degrading heme and external stimuli including ROS and inflammatory cytokines [35]. HO-1 catalyzes the breakdown of heme into three metabolic products, biliverdin (rate-limiting step), CO, and ferrous iron (Fe^2+^), by which HO-1 exerts its pharmacological activities including anticancer and anti-inflammatory effects [35,36,37].

HO-1 is a promising chemo-preventive target. Studies have shown anticancer benefits mediated by HO-1 induction in PCa models (Table 1). It has been shown that CO mediates the most beneficial HO-1 pharmacological effects involving HO-1 anticancer activities (antiapoptotic, antiproliferative, and angiogenic) and immune escape [12,37,38,39]. Several molecular mechanisms were proposed [12,13,40,41,42]. Examples of some of the proposed mechanisms include DNA damage response, G4 stability, and downstream signaling during carcinogenesis [32]. Collectively, HO-1 has been shown to prevent carcinogenesis via activation of the Nrf2/antioxidant response element (ARE) signaling pathway, protect healthy cells from oxidative stress, modulate inflammatory response, regulate cell proliferation and apoptotic cascades, and facilitate angiogenesis, indirectly mediated by its metabolic products. HO-1 is involved in modulating the mitogen-activated protein kinase pathway (MAPK) and the phosphatidylinositol 3-kinase (PI3K)/protein kinase B (Akt) pathway and signal transducer and activator of transcription 3 (STAT3) and VEGF secretion, indirectly mediated by evaluating CO and biliverdin levels [4,43,44,45]. The proposed exact mechanisms by which different kinase pathways were involved in HO-1 induction are yet to be well understood. Labanca et al. showed that transcriptional activation of HO-1 was mediated by the tumor suppressor gene, breast cancer 1 (*BRCA1*), and Nrf2 axis activation, which is a critical mechanism for the maintenance of cellular homeostasis in PCa. BRCA1 upregulates HO-1 expression in LNCap, PC3, 22RV1, and C4-2 PCa cell lines and Nrf2 cooperates with the BRCA1 protein to activate the *HO-1* gene, suggesting a new mechanism for the maintenance of cellular homeostasis in PCa [46].

## 5. HO-1 and Inflammation in Prostate Cancer

The exact molecular mechanisms of the role of the heme degradation pathway in prostate inflammation still need to be fully understood [47]. Several molecular pathways have linked HO-1 to PCa and inflammation. There are several proposed molecular mechanisms underlying the role of HO-1 in attenuating inflammation in PCa. HO-1 induction serves as a potential strategy to achieve PCa chemoprevention [39] and as an immunotherapy target and innate immune checkpoint [38]. Unresolved chronic inflammation of the prostate leads to the continuous release of proinflammatory and reactive oxygen, causing oxidative damage and initiating a carcinogenic process, which is linked to PCa growth and progression [47,48,49]. HO-1 has been shown to regulate inflammation and adaptive immunity [50]. HO-1 induction occurs in response to cellular stress and inflammation in response to diverse oxidative stimuli (adaptive response) [10,51]. HO-1 upregulation and induction correlate with reduced levels of proinflammatory cytokines and pro-angiogenic factors [13,45,52,53].

Inflammation is a critical component of PCa growth. The increased levels of circulating IL-6 in hormone-refractory PCa patients were associated with an overexpression of the activated STAT3 pathway [45,54]. HO-1 overexpression was found to inhibit PSA transcription in PCa cells and attenuate AR signaling by interfering with STAT3 signaling [45]. In in vivo studies, HO-1 induction correlates with reduced STAT3 expression in PC3 tumors, which was associated with the inhibition of tumor growth and a significant decrease in PSA. In in vitro studies, in the androgen-sensitive cell line LNCap, HO-1 induction abrogates STAT3 induction and attenuates AR signaling. HO-1 protein binds to genes involved in prostate carcinogenesis and represses IL 6 activation of the Janus kinase (JAK)-STAT pathway [45]. Taken together, HO-1 induction was associated with the downregulation of the expression of target genes associated with inflammation and angiogenesis pathways in PCa cells and xenografts [13,33,45]. Gueron et al. reported a novel molecular mechanism in which inflammatory genes were upregulated or downregulated in response to HO-1 overexpression, including matrix metalloprotease 9 [13]. HO-1 induction inhibits the activation of the AR/STAT3 and NFκB signaling pathways [45].

HO-1 has been shown to attenuate inflammation indirectly by its metabolic products acting as an anti-inflammatory and antioxidant in response to proinflammatory responses [47,55]. In the chronic prostatitis animal model, leukocyte infiltration was elevated in prostate tissue harvested from the knockout mice model lacking HO-1, suggesting HO-1 suppresses the infiltration of leukocytes into the prostate tissue after infection [47]. In this model, in CO-treated animals, CO suppresses inflammatory processes by decreasing phosphorylated MAPK extracellular signal-regulated kinase (ERK1/2) levels in the prostate samples [47]. CO inhibited IL-1β expression at the late phase in response to prostate infection by blocking inflammation and proliferation via the long-chain fatty acyl-CoA synthetase signaling pathway, suggesting suppression of chronic inflammation [47].

Chen et al. have proved that in LNCaP PCa cells, high glucose levels promoted cell apoptosis, the release of ROS, and the expression of pro-inflammatory cytokine IL-6 via inhibiting the activation of the Nrf2/ARE signaling pathway [56]. The pharmacological modulation of Nrf2/ARE by HO-1 and its bioactive compounds serve as a potential therapeutic target to suppress AR levels and sanitize PCa to anti-androgens therapy [57]. HO-1 and CO are potent antioxidants that suppress oxidative stress responses and potentially could regulate inflammation, apoptosis, cell proliferation, and angiogenesis in PCa [58]. Ferrando et al. has shown that HO-1 expression in PC3 cells activates ferrous iron and Foxo1 signaling and modulates the oxidative response in bone cells in response and adapting to environmental changes [12]. HO-1 induction upregulates genes associated with cellular adhesion in PCa cell lines and prostate tumor xenografts [59]. There are other underlying molecular mechanisms associated with the role of HO-1 in PCa. Leonardi et al. showed that HO-1 and glucocorticoid receptor (GR) interact, suggesting a crosstalk between HO-1 and GR pathways in the PC3 cell. HO-1 expression was lower under dexamethasone treatment, potentially modulating the expression of the pro-inflammatory cytokines by immune cells [60].

## 6. Induction of HO-1 by Xenobiotics PCa Models

The induction of HO-1 is a lead event in a fundamental response to pro-oxidative and proinflammatory insults in PCa cells [4]. Multiple cancer tissues have been shown to overexpress HO-1, and *HO-1* gene polymorphisms have been linked to an increased risk of developing cancer [4]. An inducible, widely expressed, pleiotropic transcription factor known as NF-κB has been linked to numerous physiological and pathological events, including infection, inflammation, and cancer. Since anti-inflammatory and anticarcinogenic drugs have the ability to activate the Nrf2/ARE/electrophile-responsive element (EpRE) cytoprotective pathways while inhibiting NF-κB signaling, alpha tocopheryl succinate (α-TOS)-induced ROS production affected the upregulation of Nrf2-driven genes leading to the suppression of NF-κB in the PCa cell line [4]. Hypoxia plays a fundamental role in PCa development, progression, and metastasis [61,62]. Several signaling pathways are activated under the hypoxic microenvironment of PCa. These pathways drive hypoxia-induced HO-1 to mediate cell adaptations and protect cells from hypoxic stress and activation of the proinflammatory pathways [18,63]. The overexpression of HO-1 is associated with better survival in the hypoxic microenvironment, suggesting a protective role of HO-1. Chronic hypoxia-induced HO-1 expression could potentially serve as a target for PCa therapy [33,64].

In PCa cell lines, α-TOS, an anticancer vitamin E derivative, enhances ROS production and lowers cell viability in a concentration-dependent way following a four-hour exposure. α-TOS increased ROS levels by twofold while decreasing the proportion of viable cells. The findings indicate that α-TOS causes oxidative stress in cell lines PC3 and LNCaP, as well as primary cultures from murine prostates [4]. Hemin, an agent used to treat porphyria-related disorders, has been implicated in the induction of HO-1. The combination of hemin and cisplatin (CIS) led to decreased NF-κB levels in PC3 cells, resulting in cell apoptosis [65]. When treating PC3 cells with hemin alone, there was a marked increase in HO-1 levels. Co-treatment of cisplatin and hemin, however, displayed a greater effect on HO-1 concentration than a singular intervention [65].

Atorvastatin, along with other cholesterol-lowering statins, has been shown to induce cell cycle arrest and apoptosis of PCa cells in both in vivo and in vitro studies through autophagy [66]. It has been postulated that this is indicative of the role of oxidative stress in the mechanistic action of this drug class in cancer treatment. In HO-1, stress response element (StRE) sites have been identified in the promotor region. When these StRE sites are activated, HO-1 levels are modulated in PC3 cells by atorvastatin [66]. These findings suggest that intervention of PCa treatment with atorvastatin has the potential to inhibit cell proliferation through the facilitation of HO-1 overexpression.

Corosolic acid (CRA), a plant-based food supplement product, has been shown to exhibit significant anti-prostate-cancer activity in TRAMP-C1 cells. Yang et al. revealed that CRA inhibits PCa growth by increasing the expression of Nrf2 and its downstream HO1 enzymes [67]. The findings suggest a potential role of CRA in PCa prevention mediated vias HO-1 signaling pathways. Camptothecin (CPT), a plant alkaloid/topoisomerase I inhibitor, possesses anticancer activities. Jayasooriya et al. show that CPT inhibited DU145 PCa cell proliferation and invasion by suppressing the NF-kB pathway and inhibiting NK-kB and pi3k/Akt activity, resulting in MMP-9 and VEGF overproduction, subsequently upregulating Nrf2-mediated HO-1 induction [68]. CPT induced HO-1 by upregulating Nr2f activity, suggesting a potential role of the Nrf2-dependent HO-1 pathway in PCa treatment [69]. Wagiel et al. showed that CO, one of the HO-1-mediated oxidation products, increased the sensitivity of PC3 cells to chemotherapy but not toward the normal cell. CO acted synergistically with doxorubicin or camptothecin to a 1000-fold increase in PCa sensitivity. CO inhibited PCa xenograft growth [70]. The researchers also showed a high nuclear expression of HO-1 in PCa cells compared to the high cytoplasmic expression of HO-1 in non-cancerous cells. Cigarette smoke induced the expression of HO-1 in PCa cells. Cigarette-smoke-induced nuclear translocation of HO-1 in DU145 and PC3 PCa cells enhanced VEGF production [44]. The results suggest that targeting nuclear HO-1 may serve as a potential new target strategy to inhibit PCa progression and angiogenesis.

**Figure 1:**
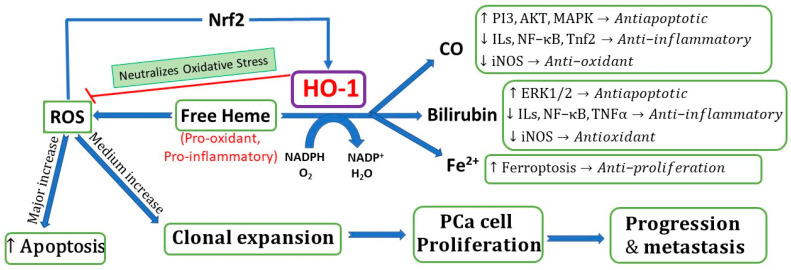
Role of HO-1 in prostate cancer pathogenesis. HO-1: heme oxygenase 1; Nrf2: nuclear factor erythroid 2-related factor 2; ROS: reactive oxygen species; Fe^2+^: ferrous iron; PI3K: phosphatidylinositol 3-kinase; MAPK: mitogen-activated protein kinase pathway; Akt: protein kinase B; IL: interleukins; NF-κB: nuclear factor kappa B; Tnf2: tumor necrosis factor-alpha promoter variant 2; iNOS: inducible nitric oxide.

Harada et al. showed that curcumin in turmeric inhibited PC3 cell viability mediated by the induction of HO-1 through the Nrf2 pathway [71]. Similarly, Xu et al. reported that an upregulation of HO-1 expression was primarily mediated by the Nrf2-dependent pathway. The researchers demonstrated that treatment with phenethyl isothiocyanate (PEITC) induced ARE-driven *HO-1* gene expression in PC3 cells [72]. Zhou et al. showed that HO-1 induction retarded the growth of LNCaP cells. HO-1 induction inhibited xenotropic murine leukemia virus infection of the LNCaP cell [73]. HO-1 induction downregulated annexin 2 (ANXA2) in PC3 PCa and bone metastasis cells [74]. These findings suggest that HO-1 may prevent PCa cell invasion into other surrounding organs. Cascardo et al. showed that HO-1 induction by hemin caused a significant shift in the cellular metabolism of PC3 and C4-2B. HO-1 impairs the metabolic status of PCa cells via modulating aerobic glycolysis, which results in a less aggressive form of PCa [75]. Keum et al. have shown that the oral administration of broccoli sprouts to TRAMP mice exhibited a significant inhibition of PCa growth [76]. The data revealed that HO-1 induction was associated with an increase in Nrf2 expression and the upregulation of apoptotic proteins. These findings suggest the potential inhibitory effects of dietary broccoli sprouts on PCa carcinogenesis. Acquaviva et al. showed the beneficial effects of Oleuropein, the olive extract, in PCa cell lines. Oleuropein inhibits LNCaP and DU 145 PCa cell proliferation and induces apoptosis potentially via the induction of pAkt and HO-1 [68]. Vallelian et al. showed that ellagic acid (EA) induced apoptosis through the activation and inactivation of several pathways including downregulating HO-1 expression and increasing ROS levels and activating caspase 3. EA caused a significant decrease in HO-1 expression in LNCaP-treated cells [77]. Zhang et al. reported that exosomes, which are important mediators of cell–cell interactions, promoted PCa cells to advanced-stage CRPC. Exosomes in the PCa microenvironment resulted in PCa progression to androgen-independent mediated via the upregulation of HO-1. After treatments with PC3 exosomes, HO-1 proteins and mRNA levels were significantly upregulated [78]. These results suggest that exosomes may play an important role in the upregulation of HO-1 in CRPC. Saski et al. reported that HO-1 expression was induced by sodium nitroprusside (nitric oxide donor) in DU145 [79]. However, HO-1 antisense S-oligodeoxynucleotide treatment increased NO-induced growth inhibition, suggesting the crosstalk between NO/HO-1 pathways (Figure 1).

## 7. Inhibition of HO-1 by Xenobiotics in PCa Models

Alaoui-Jamali et al. developed a potential selective HO-1 inhibitor with the intention of facilitating tumor suppression. OB-24, a molecule synthesized from the imidazole class, demonstrates antitumor and antimetastatic activity in human refractory PCa cells (HRPCA) [30]. Clinical studies found that OB-24 has a similar inhibitory effect on HO-1 activity as shRNA in PCa cells. shRNA with HO-1 was observed to reduce the activation of MAPK ERK and p38 kinase. MAPK ERK signaling cascades phosphorylate proteins, leading to their activation or inactivation. Incessant MAPK activation is associated with many malignant tumors. Experimental results of OB-24 exposure demonstrated that PC3M cells showed between 20 and 60% cell line and growth inhibition following introduction of more than 5.5 µmol/L of OB-24 [30]. In contrast, single chemotherapeutic agents alone showed insignificant stimulation of HO-1 cells. The results of these clinical findings imply that treatment with this selective inhibitor alone is beneficial in that it notably reduces tumor growth in mice with high-risk PCa [30]. Combination therapy involving the current chemotherapy drugs, Taxol, and the specific targeting of tumorigenesis-promoting pathways indicates an exemplary use of OB-24 in this field.

As HO-1 shows an incidence of overexpression in PCa, recent studies have chosen to focus on the various receptors and pathways responsible. In a study conducted by Romeo et al., it was observed that sigma receptors, non-G protein-coupled receptor (GPCR) transmembrane proteins, are expressed in various cancer cell lines. Their two subtypes, σ-1R and σ-2R, are, respectively, antagonized and agonized to promote cancer inhibitory effects [80]. When exposing DU145 cells to sigma receptor ligands, it was found that cell proliferation was noticeably reduced by approximately 50%. When used in combination with HO-1 receptors, it was found that exposure to 10 μM of both the ligand and inhibitor greatly reduced proliferation by 75% [80]. This response suggests that while targeting the use of either an inhibitor or sigma receptor is effective in the reduction in cell viability, the greatest effect comes from multi-targeted combination therapy. The implications of this study include exploring the mechanisms and signaling pathways of this disease, which may be vital in working toward a cure.

Aside from the multifactorial combination of current chemotherapy drugs and selective HO-1 inhibitors, various medications have been implicated as potential cancer treatments. One such drug is metformin. Metformin is a biguanide that is typically used in the treatment of type II diabetes. It is used to control blood glucose levels by reducing the liver’s ability to release stored glucose. Metformin was determined to have antiproliferative effects regarding cancer, which is associated with mitochondrial respiration inhibition and cell cycle arrest [81]. Since cancer cells rely on glucose for energy more than normal cells, studies show that metformin’s antiproliferative activity is dependent on glucose concentration. In the instance of PCa, metformin was found to suppress in vitro cancer growth and oxidative stress [81]. When used in tandem with HO-1 inhibitors, ROS levels decreased. These findings indicate that the coordinated effect of this multidrug treatment has a profound influence on PCa cells. Using metformin to treat patients with a diagnosis of PCa can induce cell apoptosis and encourage glucose deprivation. Future research should focus on the long-term effects of combined metformin and HO-1 inhibitor use and the likelihood of recurrence if remission were to occur.

Polyphenolic compounds demonstrate antioxidant and chemopreventive properties by neutralizing free radicals in the body, leading to a reduction in cancer risk and progression and oxidative side effects on biological molecules. Oleuropein, a compound found in olive leaves, has been observed to possess health-promoting properties, including those that are anti-inflammatory. Acquaviva et al. reported that both in vivo and in vitro studies have found that oleuropein has antiangiogenic properties and the ability to inhibit tumor growth [68]. After exposure to 100–500 μM oleuropein for 72 h, PCa cell lines DU145 and LNCaP displayed significant diminishment in cell viability [68]. In a similar study, EA, an organic polyphenol found in fruits and vegetables, had a similar effect on both LNCaP cells and HO-1. After treatment with 25–50 μM of EA, LNCaP cells displayed a prominent decrease in HO-1 fluorescence via Western blot analysis [34]. The results of these studies suggest that the PCa antiproliferative effects of naturally occurring phenolic compounds are potentially mediated through the inhibition of HO-1 activity. To further understand the role of phenolic compounds in cancer treatment, additional mechanistic studies involving HO-1 are required.

Romeo et al. have shown that targeting both HO-1 pathways may serve as a novel therapeutic strategy in PCa therapy [80]. The simultaneous targeting of HO-1 and sigma receptor proteins reduces DU145 cell proliferation. Mucha et al. have investigated a novel HO-1 inhibitor, imidazole-based inhibitors (SV-11199), in the DU145 PCa cell line [22]. The inhibition of HO activity by SV-11199 was associated with a decrease in cell viability and increased sensitivity of PCa to chemotherapy.

## 8. Does HO-1 Have Pro-Cancerous or Anti-Cancerous Effects in PCa?

There are conflicting reports on whether HO-1 facilitates PCa growth and metastasis or exhibits anti-prostate-cancer effects, and contrasting data are reported. Herein, we presented the current understating of the role of HO-1 in PCa. HO-1 is involved in multiple cellular signaling pathways. In normal cells, HO-1 acts as a cytoprotective enzyme against oxidative stress, exhibiting cytotoxicity and anti-inflammatory effects, which can result in cancer prevention. In DNA-damaged cancer cells, HO-1 halts pro-carcinogenic activation mediated by ROS (Figure 1). The available evidence suggests that the pharmacological effects of HO-1 largely depend on the amount of cellular iron and ROS, HO-1 expression levels, and the stages of PCa. As summarized in Figure 1, HO-1 responds to stimulants that generate high levels of ROS differently to medium increases in ROS. The amount of cellular iron and ROS will determine the role of HO-1 in PCa. HO-1 overexpression can increase ferrous-amplified oxidative stress, resulting in cell ferroptosis [82], suggesting the complex role and crosstalk between HO-1 and ROS systems (Figure 1). A major increase in ROS enforces HO-1 to a protective role and leads to the activation HO-1 pathways through antiapoptotic activities, which suppress tumor growth. On the other hand, a medium increase in ROS enforces HO-1 to the perpetrator role, which can increase cancer survival and treatment resistance, and the metastatic potential (Figure 1).

ROS (pro-oxidant) can regulate cell proliferation and apoptosis based on its low or high levels [83,84]. Low ROS levels promote cell survival (suppressed proliferation), and high ROS promotes cell death (rapid reaction) [84]. As illustrated in these reports, a moderate increase in ROS promotes PCa carcinogenesis, growth, and survival/metastasis in various PCa cell lines. However, a major increase (substantial increase) in ROS levels can oxidize vital molecules within PCa cells, causing HO-1 (antioxidant) to suppress the transformation of PCa [82,83,84]. Under oxidative conditions, ROS-induced increase in HO-1 thereby protects PCa cells by scavenging ROS and neutralizing ROS-mediated oxidative cell damage [82]. Following activation of the Nrf2 pathway, it promotes the transcription of antioxidant HO-1 and reduces ROS-induced oxidative stress and damage [82,83,84]. The overexpression of HO-1 as a result of induction by ROS was reported to enhance the survival of PCa cells via an increase in antiapoptotic proteins and by promoting autophagy [82,85].

The available evidence in the literature is summarized in Table 2, which reveals contradictory results. The inducible HO-1 exhibits anticancer inhibitory effects mediated by CO, bilirubin, and Fe^+2^ metabolic products via the activation of multiple signaling pathways, including kinase pathways PI3K/Akt, MAPKs, Nrf2/ARE, ERK1/2, and Akt/mTOR, and ferroptosis induction, and the upregulation of apoptotic proteins, which neutralize oxidative stress, can lead to the inhibition of PCa proliferation and the induction of apoptosis (Figure 1). Targeting HO-1 signaling pathways by xenobiotics could provide a promising novel PCa therapeutic opportunity. Some evidence suggests that the pharmacological inhibition of HO-1 demonstrated a promising approach to inhibiting PCa growth and metastasis. The combination of the HO-1 inhibitor with chemotherapy produced a synergistic anticancer activity [30].

**Figure 2:**
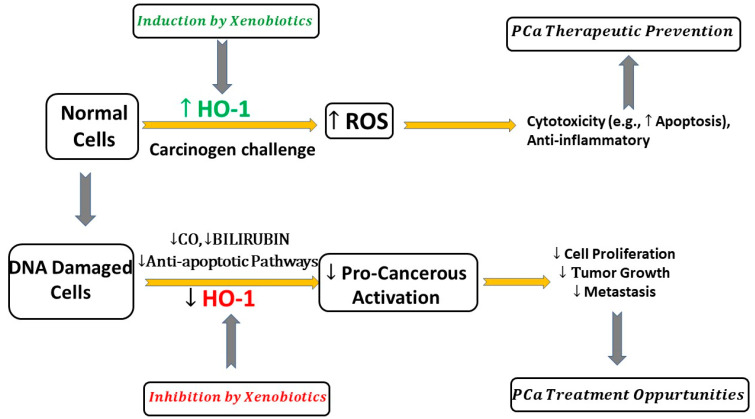
Effect of HO-1 modulators on prostate cancer development and progression. HO-1: heme oxygenase 1; ROS: reactive oxygen species; CO: carbon oxide.

A review conducted by Nitti et al. on the regulations of HO-1 expression and its clinical significance in cancer therapy suggests HO-1 is a promising biomarker for cancer progression [9]. Though there is limited information on microRNA (miRNA)-mediated regulation of HO-1 in PCa, miR-193a-5p lowers the expression of HO-1, facilitating docetaxel-related PCa cell apoptosis [86], Cheng et al. summarized the current literature on the role of microRNA in the post-transcriptional regulation of HO-1 expression indirectly through modulating the Nrf2/ARE pathway in different cancer types [87]. Another review by Hoang et al. highlighted an interesting new immunotherapy approach via boosting anticancer immunity by targeting HO activity [11]. In the context of PCa, our findings that many preclinical and clinical studies highlight the correlation between HO-1 expression and PCa progression and poor clinical outcomes (summarized in Table 1) are consistent with the role of this stress protein in the prevention and treatment of cancer. Figure 2 summarizes a range of possible molecular mechanisms mediated by HO-1 induction or inhibition in PCa.

## 9. Conclusions

The literature reflects opposing roles of HO-1 in PCa. Our analyses, as summarized in Figure 2, demonstrate that the amount of cellular ROS can determine the role of HO-1 in PCa. In a normal cell, HO-1 induction can act as a cytoprotective protein by neutralizing the oxidative stress, which leads to cell death and PCa growth inhibition. However, in PCa, HO-1 inhibition by xenobiotics tilts the balance to promote apoptosis and inhibit PCa proliferation and metastasis. HO-1 may serve as a potential new target for therapeutic prevention by increasing HO-1 functions in normal cells, while also facilitating treatment by inhibiting PCa progression and angiogenesis.

## Figures and Tables

**Table 1:** Expression of HO-1 in different prostate cancer models.

Model	HO-1 Increase/Decrease	Signaling Proteins	Significance	Ref
Normal, human PCa samples	increase	Heme molecule (Fe-protoporphyrin IX) cleaved by HO-1 → biliverdin	↑ HO-1 expression in BPH and in PCa tissues	[21]
Rat PCa tissues prostate tumors carrying Dunning G (G) cells poorly metastatic AT-1 cells metastatic MatLyLu cells	increase	HO-1 mRNA and proteins were found to be expressed in rat prostate tumors, non-malignant prostate tissue, as well as in macrophages.	↑ HO-1 expression in rat PCa model	[27]
Human primary PCa tissues and human prostate bone metastases	increase	Small metastatic tumors were considerably more effective in attracting HO-1+ macrophages than larger non-meta-static ones. ↑ accumulation of HO-1+ macrophages at the invasive tumor front, and at high-grade tumors → bone metastases	Selective knockout of HO-1 in macrophages reduced PCa growth and metastatic capacity in animals. Potential role of the extratemporal HO-1+ macrophages in PCa.	[27]
Human PCa and BPH samples	increase	HO-1 is expressed in the nuclei of PCa cells in patient specimens. ↑ in HO-1 expression was found in the cytoplasm of PCa cells, in epithelial cells of adjacent non-neoplastic areas, and in epithelial cells of BPH.	HO-1 cytoplasmic localization is similar in clinical PCa, non-neoplastic surrounding parenchyma, and BPH.	[28]
PC3 and LNCaP cell lines	increase	↑ HO-1 expression	Hemin can induce nuclear translocation of HO-1 in PCa cells.	[28]
PC3 xenografts	increase	↑ in HO-1 expression was found in positive macrophages in PCa, in the tumor PC3 microenvironment. HO-1-derived CO targets STAT3 and mitochondrial pathways to control EMT; expression of E-cadherin in cancer cells.	HO-1 modulates PCa progression HO-1 in macrophages and controls immune cell infiltration to the tumor microenvironment.	[14]
PC3HO-1 xenografts	increase	↑HO-1 overexpression → ↓VEGFA, VEGFC, HIF1α and α5β1 integrin (Inflammatory and pro-angiogenic) ↓ NF-κB-mediated transcription	↓ neovascularization ↓ VEGFR2 expression HO-1 may regulate angiogenesis	[33]
Human plasma HO-1 level in PCa patients	increase	Lack of correlation between HO-1 and vascular damage	There were no correlations between HO-1 levels with VEGF or Gleason stage.	[29]
PC3, LNCaP, and MDA PCa2b. PC3HO-1 xenografts	increase	↓ MMP-9 production and activity Hemin ↑ HO-1 mRNA	PCa cell proliferation, invasion, and migration were significantly reduced.	[13]
PCa patient samples PC3 xenografts HO-1 knockout mice	increase	↑ HO-1 expression in metastatic PCa samples and the tumor microenvironment → effects on PCa progression	HO-1 may have a significant role in PCa progression via regulation of mitochondrial activity and expression of E-cadherin.	[15]

PCa: prostate cancer, HO-1: Hem oxygenase-1, PC3HO-1: intradermal inoculation of PC3 cells stable transfected with HO-1, BPH: benign prostatic hyperplasia, STAT3: signal transducer and activator of transcription 3, EMT: epithelial–mesenchymal transition, NF-κB: nuclear factor kappa B, VEGFA: vascular endothelial growth factor A, MMP-9: matrix metallopeptidase-9.

**Table 2:** Agents modulating HO-1 expression and/or activity in PCa.

Agent	HO-1 Increase/Decrease	PCa Model	Pathway Affected	Significance	Ref
γ-tocopherol succinate	Increase	PC3	↓ NF-κB activation ↑ Nrf2 expression and activity, ↑ HO-1 expression	↓ PCa cell viability and ↑ selective cytotoxicity via oxidative stress.	[4]
OB-24	Decrease	LNCap, VCap, DU145 PCa human samples	↓ MAPK/ERK pathway in HO-1 activity	HO-1 downregulation prevented PCa progression. Treatment of mice with OB-24 drastically reduced tumor size.	[30]
Metformin + VP1347	Decrease	DU145	Cell Cycle	Combination of HO-1 inhibitor and metformin use leads to glucose deprivation and ROS decrease.	[81]
Atorvastatin	Increase	PC3	Activation of the StREs of HO-1 promotor	The upregulation of HO-1 expression led to inhibition of cell proliferation and invasion.	[66]
Hemin + Cisplatin (CIS)	Increase	PC3	NF-κB and iNOS	Combination of hemin and cisplatin leads to decreased NF-κB levels in PC3 cells, resulting in cell apoptosis.	[65]
Corosolic acid	Increase	TRAMP-CA PCa	↓ Nrf2	CRA inhibits TRAMP-C1 cell growth and induced the expression levels of Nrf2, HO-1, and NQO1.	[67]
Camptothecin	Increase	DU145 cells	↓ Nrf2/HO-1 pathway ↓↓ NF-κB MMP-9 ↓ VEGF production ↓PI3K/Akt nuclear factor-kB (NF-κB) activity.	CPT inhibits PCa growth and invasion.	[69]
CO	Increase	Human PCa samples PC3 xenografts	↓ nucleotide and amino acid synthetic pathways Cell cycle arrest	CO inhibits PCa growth	[70]
Cigarette smoke	Increase	DU145 PC3	↑ VEGF secretion ↓ PCa grwoth	↓ PCa cell proliferation and migration	[44]
Curcumin	Increase	PC3	Nrf2/HO-1 pathway	↓ Cell proliferation	[71]
Hemin ± calcitonin	Increase	LNCaP infected with XMRV	HO-1 host defense against retrovirus infection	↓ Cell proliferation	[73]
ANXA2 + HO-1	Increase	PC3 Osteoclast PCa PCa human samples	↑ ANXA2/ANXA2-R expression	↓ PCa bone metastasis ↓ PCa invasion	[74]
Hemin	Increase	PC3 C4-2B	↓ glucose consumption ↓ ATP production	↓ PCa metabolism Less aggressive form of PCa	[75]
PEITC	Increase	PC3	↑ Nrf2 accumulation ↑ ERK12, JNK1/2 phosphorylation activities	↓ PCa cell viability ↑ ARE activity	[72]
Broccoli sprouts	Increase	PCa Male TRAMP mice	↑ Nrf2/ARE signaling pathways ↑ Keap1 proteins ↑ Apoptosis markers ↑ cleaved Caspase 3, Bax, Bcl-XL proteins ↓Akt/mTOR signaling	↓ PCa growth	[76]
Oleuropein	Increase	LNCaP DU145	↑ pAkt	↓ PCa cell viability	[68]
Ellagic acid	Decrease	LNCaP	↓ mTOR signaling, ↓ SIRT1, HuR expression ↑ ROS ↑ p21 expression ↓ IL6 levels	↑ PCa apoptosis ↓ Inflammation	[77]
HO-1 inhibitor LS/0 LS4/28 LS6/42	Decrease	DU145	Targeting HO-1 signaling pathway	↓ PCa cell viability ↓ PCa cell proliferation	[80]
HO-1 inhibitor imidazole-based inhibitor (SLV-11199)	Decrease	DU145	SLV-11199 decreased cell migration and inhibited MMP-1 and MMP-9 expression ↑ sensitization to chemotherapy	↓ PCa cell viability	[22]
Exosomes	Increase	PC-3 derived exosomes cells	↑ HO-1 proteins and mRNA expression	↑ CRPC progression	[78]
Sodium nitroprusside (NO donor)	Increase	DU145	↑ HO-1 Expression ↑ Bcl-2 expression	↓ PCa cell viability	[79]

ERK: extracellular signal-regulated kinase; MAPK: mitogen-activated kinase; ARE: antioxidant response element; StRE: stress response element; NQO1: quinone oxidoreductase-1; MMP-9: matrix metalloproteinase-9; VEGF: vascular endothelial growth factor; PI3K/Akt: phosphoinositide 3-kinase; NF-κB: nuclear factor-kappa B; CO: carbon monoxide; xmrv: xenotropic murine leukemia virus-related virus; ANXA2: annexin; PEITC: phenethyl isothiocyanate; SIRT1: silent information regulator 1; HuR: human antigen R; CRPC: castration-resistant prostate cancer; NO: nitric oxide.

## Data Availability

Data sharing not applicable.
